# Supplementary material for: High Incidence of Adverse Outcomes in Haemodialysis Patients with Diabetes with or without Diabetic Foot Syndrome: A 5-Year Observational Study in Lleida, Spain
Source: J Clin Med. 2021 Mar 26;10(7):1368. doi: 10.3390/jcm10071368 (PMC8037880; doi:10.3390/jcm10071368)
Supplement: Supplementary file 1 [file jcm-10-01368-s001.pdf]

Table S1. Diabetes-related microvascular complications recorded in the populations studied.

Variable	All HD patients	Patients without DM (N=135)	Patients with DM (N=85)		P-value No DM vs. DM	P-value No DF vs. DF
			No diabetic foot* (N=55)	Diabetic foot (N=30)		
**Nephropathy, n (%)**						
Diabetic nephropathy	85 (38.6)	0.0 (0)	55 (100)	30 (100)	<0.001	0.259
Other causes	135 (61.4)	135 (100)	0 (0.0)	0 (0.0)	-	-
**Retinopathy, n (%)**					<0.001	0.062
No retinopathy	138 (62.7)	128 (94.8)	7 (12.7)	3 (10.0)		
Diabetic	-	-	31 (56.4)	24 (80.0)		
Unknown	-	-	17 (30.9)	3 (10.0)		
**Neuropathy, n (%)**					<0.001	1.000
No neuropathy		126 (93.3)	1 (1.8)	0 (0.0)		
Diabetic	82 (37.3)	-	53 (96.4)	29 (96.7)		
Unknown	-	-	1 (1.8)	1 (3.3)		

Figure S1. Kaplan-Meyer survival curves in patients with DM vs. those without regarding the clinical outcomes studied.

All-cause mortality

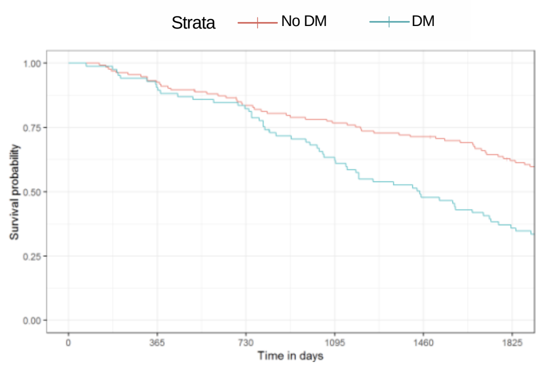

CV mortality

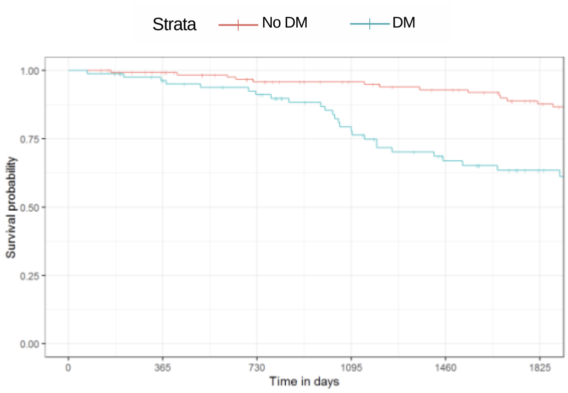

CV event

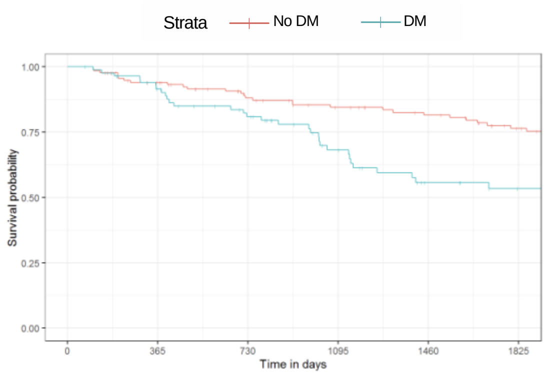

MACE

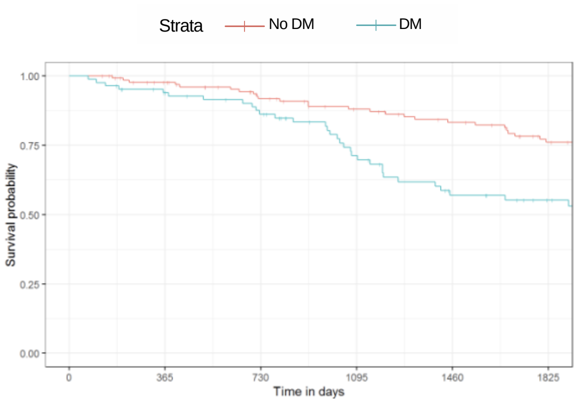

New ulcer or amputation

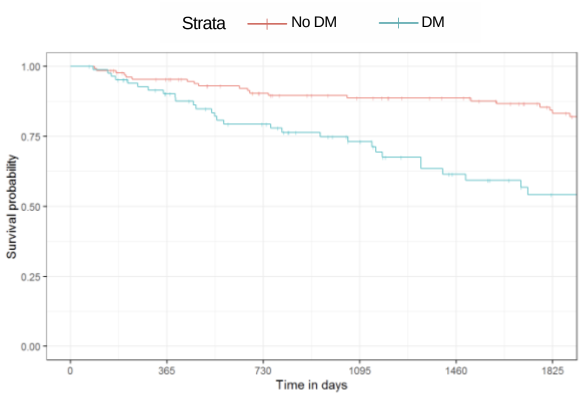

CV, cardiovascular; HR, hazard ratio; MACE, major adverse cardiovascular event

Figure S2. Kaplan-Meyer survival curves in patients with DM comparing those with and without prior diabetic foot

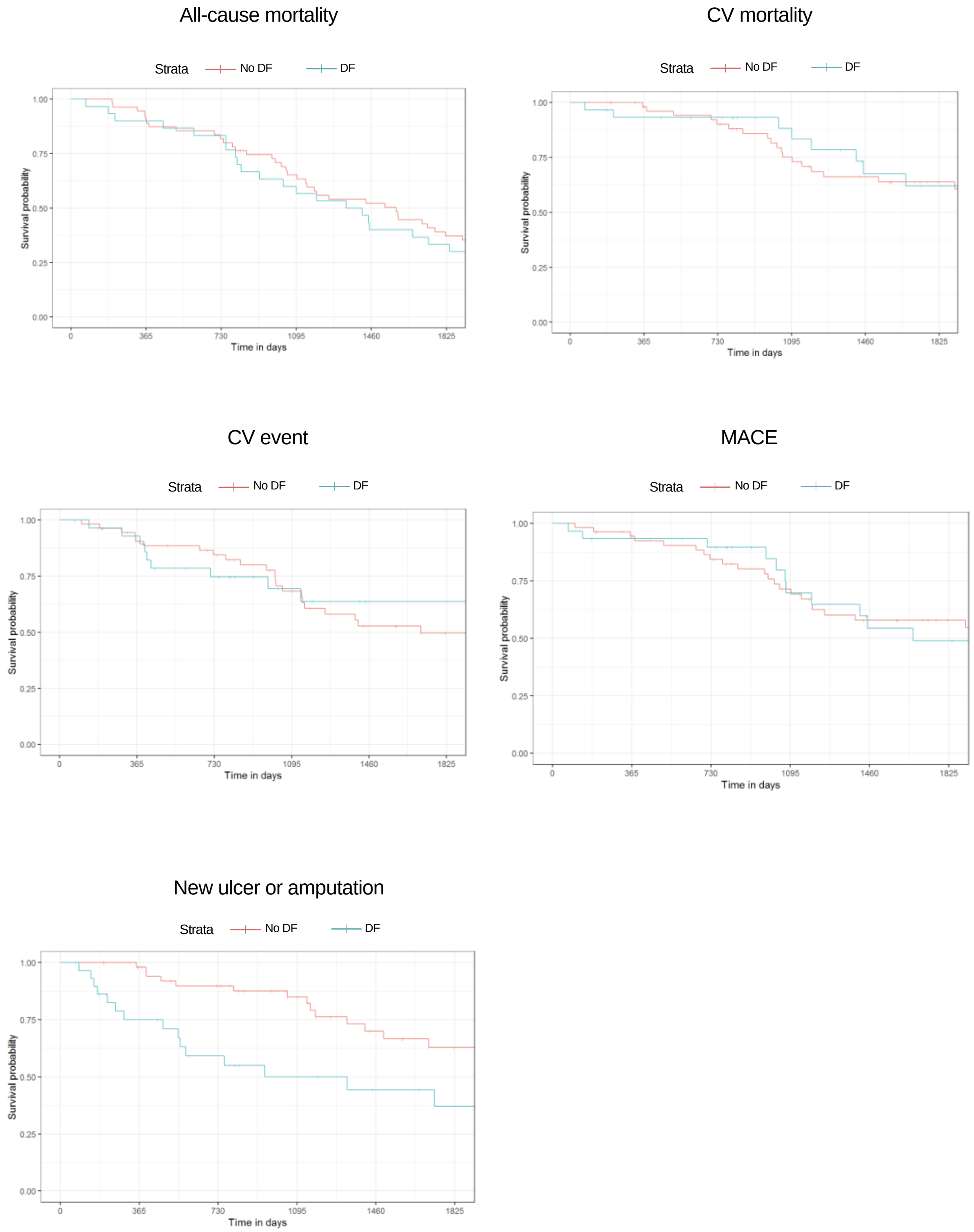

CV, cardiovascular; HR, hazard ratio; MACE, major adverse cardiovascular event
